# Gender differences in autism prevalence: origins of bias and its current scientific relevance

**DOI:** 10.3389/fpsyt.2026.1739570

**Published:** 2026-07-10

**Authors:** Patricia Peña-Casquero, Dominika Zofia Wojcik, Ricardo Canal-Bedia

**Affiliations:** 1 University of Salamanca, Salamanca, Spain; 2 Instituto Universitario de Integración a la Comunidad (INICO)-InFoAutismo, Salamanca, Spain; 3 Instituto de Investigacion Biomédica de Salamanca (IBSAL), Salamanca, Spain

**Keywords:** autism spectrum disorder, clinical implications, diagnosis, equity, gender bias, gender-diverse individuals, inclusion, male-centered model of autism

## Abstract

The diagnosis of Autism Spectrum Disorder has historically been biased toward a male-centered model, rendering women and gender-diverse individuals largely invisible. This commentary reviews the origins of such bias, its contemporary manifestations, and the clinical, social, epistemological, and mental health equity implications it entails. Pathways toward a more equitable and inclusive model are proposed.

## Historical roots of a male-centered diagnostic model and its impact on equity

Autism Spectrum Disorder (ASD)^1^ refers to a neurobiological condition characterized by difficulties in reciprocal social communication and interaction, along with restricted, repetitive, and stereotyped patterns of behavior, interests, or activities (1). Although autism has historically been identified four times more frequently in males than in females (2), some recent large-scale population-based studies have raised the possibility that sex differences in autism prevalence may be smaller than traditionally reported. For example, a nationwide longitudinal study in Sweden found that, although boys are diagnosed earlier, the proportion of ASD diagnoses in males and females becomes nearly equivalent by early adulthood, suggesting a convergence toward a 1:1 ratio (3). This “catch-up” effect has been interpreted as evidence that females may be underdiagnosed or diagnosed later due to differences in clinical presentation and potential diagnostic biases. Accordingly, these findings support the view that the true prevalence of ASD may be more balanced across sexes than suggested by childhood diagnosis rates, although this interpretation remains under debate. In this context, Burrows et al. (4) reported a near 1:1 sex ratio within a subgroup of children showing high and stable social communication difficulties across development. Conversely, among individuals with both autism and intellectual disability, the ratio is closer to 2:1 (5). The most recent prevalence data reported by the Spanish-language website of the U.S. Centers for Disease Control and Prevention (CDC Español) estimates autism at 1 in 36 children (significantly higher than the 2018 estimate of 1 in 44) with a ratio of 4.2 males for every female (6). We summarize the prevalence findings in **Table 1**.

**Table 1:** Overview of male/female prevalence studies.

Country/study	ASD Prevalence	Male: female ratio (ASD)	ASD + ID (%)	Male: female ratio (ASD + ID)	Notes
General estimate (2)	—	~4:1	—	—	Historically reported ratio
Meta-analysis (7)	—	~3:1	—	—	Adjusted estimate accounting for ascertainment bias
USA (6)	1 in 36	~4.2:1	~30–35%	~2:1	Most recent large-scale prevalence estimate
Sweden (3)	~1–1.5%	~3:1 (childhood) → ~1:1 (adulthood)	~25–30%	~2:1	Evidence of “female catch-up” effect
Burrows et al. (4)	—	~1:1 (symptom subgroup)	—	—	Symptom trajectories in at-risk children; no confirmed ASD diagnosis
USA (5)	—	—	—	~2:1	ASD with co-occurring intellectual disability

CDC, Spanish-language Center for Disease Control and Prevention); ASD, Autism Spectrum Disorder; ID, Intellectual Disability; USA, The United States of America.

The average age of diagnosis remains stable (8), occurring around age 5 for children without co-occurring intellectual disability or with milder symptoms, and approximately 67 months for those with cognitive impairment or more severe forms of autism (9). However, a growing number of individuals receive diagnoses much later in life, often during adolescence or adulthood, a phenomenon referred to in the literature as “late diagnosis” (10).

Several studies suggest that delayed diagnosis occurs more frequently among women (11), partly due to differences in symptom presentation and the use of compensatory strategies such as social camouflaging, which may mask autistic traits during childhood and adolescence (12–14). This diagnostic delay is particularly relevant because it may limit early access to appropriate support and interventions, while increasing the risk of misdiagnosis and unmet mental health needs. Despite the growing recognition of this phenomenon, there is still no consensus on the precise definition of “late diagnosis” in autism (15). These discrepancies have not only been normalized but also sustained for decades within both research and clinical practice. Most early studies and diagnostic criteria were developed almost exclusively on male and child samples, thereby consolidating a male-centered clinical model of autism (16). This biased perspective has contributed to overlooking the ways in which ASD may manifest in girls and women, who often present symptoms that are less disruptive or more socially acceptable. In many cases, autistic girls and women engage in social camouflaging, i.e., a set of conscious or unconscious strategies used to mask autistic traits and adapt to social expectations (17). These strategies may include rehearsing conversational scripts, imitating peers, social behaviors, suppressing repetitive movements, forcing eye contact during interactions, or closely observing and copying social norms (18, 19). While masking may facilitate short-term social adaptation, it can also contribute to diagnostic invisibility and delayed recognition of autism (20).

The lack of scrutiny toward this model can be explained by a combination of factors: gender biases in medical and psychological research, the absence of diagnostic tools sensitive to female profiles, and sociocultural norms that trivialized autistic girls’ behaviors as mere personality traits—such as shyness, sensitivity, or perfectionism (21). As a consequence, there have been generations of women who either remained without an accurate diagnosis or received misdiagnoses such as anxiety, depression, or personality disorders which has affected their mental health and personal development (22).

In this context, it becomes necessary to question why this gap has been normalized for so long across clinical, educational, and scientific systems, as well as to consider the repercussions this omission has had on diagnostic equity and access to appropriate support.

Beyond documenting disparities, this paper advances a central argument: gender bias in autism diagnosis should not be understood merely as a limitation of clinical practice, but as a structural constraint embedded in the historical and epistemological construction of autism itself. The predominance of a male-centered model has not only shaped diagnostic tools and research priorities but has also defined what is recognized as “autistic,” thereby systematically excluding or misinterpreting non-male presentations. From this perspective, diagnostic inequities are not incidental but constitutive of the current framework.

## Historical context of autism diagnosis

Although several early studies have mentioned autism in the context of the schizophrenia spectrum (23), the concept of autism formally emerged in the 1940s through the pioneering work of Leo Kanner in the United States and Hans Asperger in Austria. Kanner described eleven children (three of them girls) who shared common characteristics such as social withdrawal, literal language, and repetitive behavior patterns, coining the term “early infantile autism” (24). Asperger, in turn, observed similar traits in a group of children (all boys) with relatively preserved verbal abilities and a peculiar cognitive style, which later came to be known as Asperger’s syndrome (25). Although Kanner and Asperger described predominantly male cases, it was the subsequent institutionalization of their work within diagnostic systems and research traditions that contributed to consolidating a predominantly male-centered model of autism. This limitation was reinforced in early classification systems, such as the DSM-III (26) and DSM-IV (27), which were constructed almost exclusively from research conducted with male populations.

As a result, diagnostic criteria came to reflect a “typical presentation” of male ASD, characterized by socially withdrawn behavior, highly restricted and unusual interests, difficulties in both verbal and nonverbal communication, and overt repetitive behaviors. This structural bias persisted for decades and became widely normalized in clinical and educational practice, reinforcing the notion that autism was predominantly a male disorder (28). Though this notion has since been challenged, as we aim to demonstrate, the idea still prevails in some contexts today, disproportionately affecting women and gender-diverse individuals on the spectrum.

## Emergence of the gender issue

Between the 1980s and 2000s, studies began to highlight the consolidation of gender bias in ASD diagnosis. Lorna Wing introduced Asperger’s syndrome into English-language literature. Although she described female cases as well, the condition continued to be regarded as predominantly male (29). At the same time, epidemiological studies such as the Camberwell study (30) showed that diagnoses in autistic girls were more frequently associated with intellectual disability, pointing to a systematic underrepresentation of cases without intellectual disability.

In the early 2000s, studies emerged suggesting that autism represents an extreme form of the “male brain,” characterized by a greater tendency toward systematizing and a reduced capacity for empathy (31). Although this hypothesis was criticized for relying on biologically deterministic notions of gender and for failing to adequately explain the female presentation of autism, it stimulated subsequent research with a broader focus, including neuroimaging studies, analyses of social camouflaging, and behavioral manifestations in autistic adults (28).

During this period, the first European projects such as *Autism in Pink* were launched, aimed at investigating the experiences and specific needs of autistic adult women (32). In parallel, the first gender-focused studies introduced the concept of “camouflaging” or social masking. Key research on gender differences in autism examined this phenomenon, as well as the existence of atypical female phenotypes (18, 33, 34) and research also began to explore adult diagnosis, highlighting both the impact of camouflaging and the high psychological costs of late diagnosis (12).

Building on these findings, new studies began to question the reliability of the 4:1 ratio (7). This view has been challenged by more recent work showing diagnostic bias linked to the different clinical presentations of women and non-binary individuals, who frequently rely on camouflaging or social masking—conscious or unconscious strategies to conceal autistic traits and adapt to social expectations (13).

Recognition of camouflaging has led to questioning the validity of the traditional male-to-female diagnostic ratio and to the proposal of a female autism phenotype. This profile, described as a combination of compensatory strategies, superficial social adaptation, and internalized symptoms, has helped bring visibility to some of the ways autism may manifest in women and to the substantial heterogeneity observed in female presentations. However, these traits are often misinterpreted or overlooked in conventional clinical assessments (13). Contemporary research has shown that this profile directly influences diagnostic delay, with women often receiving a diagnosis several years later than men.

Moreover, autistic individuals have been found to display greater diversity in gender identity (35) and less adherence to traditional gender roles (36). They also more frequently report non-binary identities or incongruence with their assigned sex at birth (37, 38). Overrepresentation of transgender individuals among those with autism, and vice versa, has also been documented (39), suggesting that the social and communicative characteristics of autism may contribute to weaker identification with gender norms, thereby fostering greater diversity in identities.

Recent research suggests that gender differences in autism may be influenced by social expectations and stereotypes that shape both the expression of symptoms and their clinical recognition (40). For example, socially withdrawn behavior in boys may raise concerns about developmental disorders, whereas similar behaviors in girls are often interpreted as shyness or emotional sensitivity. Likewise, restricted interests in girls—such as literature, animals, or popular culture—may be perceived as socially typical and therefore overlooked during clinical evaluation. Individuals assigned female at birth, as well as those identifying as female or wishing to be recognized as gender diverse, are diagnosed with ASD much later than those assigned male at birth, those identifying as male, or cisgender individuals, (41). This indicates that disparities persist regardless of the dimension considered (assigned sex, gender identity, or gender diversity), suggesting that each dimension contributes differently to diagnostic delay.

This relationship has led to the proposal that certain characteristics of autism—for example, less attachment to social conventions—may foster a more flexible experience of gender (42). This raises the need for clinical and research approaches that integrate the intersection between neurodiversity and gender diversity, avoiding normative models that render non-hegemonic identities invisible. In practice, this may involve developing assessment frameworks that consider both sex-related biological factors and gender-related sociocultural experiences. For example, diagnostic protocols could include measures of camouflaging, gender identity, and social experiences linked to gender norms in order to better understand how autistic traits may manifest across different gender identities.

In addition, recent neuroscience studies have provided evidence of differences in cortical development patterns and neurocomputational profiles between males and females within the spectrum, suggesting that genuine neurobiological differences may coexist with clinical disparities (43). Consequently, several authors emphasize the urgency of ensuring that clinical and biomedical studies include gender-balanced samples to prevent the perpetuation of inequalities in autism detection and care (44). Recording sex assigned at birth and gender identity separately would allow researchers to examine whether diagnostic timing, symptom presentation, or camouflaging strategies differ according to biological sex, gender identity, or their interaction.

These converging findings highlight the persistence of structural inequities in diagnostic frameworks, leading to renewed scrutiny of gender-related disparities in autism research and practice. The apparent male predominance in ASD is not solely an epidemiological reality but is also shaped by diagnostic practices, gendered social expectations, and camouflaging behaviors, all of which contribute to the systematic underrecognition of diverse autistic presentations across gender identities. Consequently, a comprehensive understanding of autism requires integrating biological, clinical, and sociocultural dimensions in order to adequately account for both diagnostic disparities and the heterogeneity of autistic phenotypes.

## Why is it important to explore this difference?

**Figure 1:**
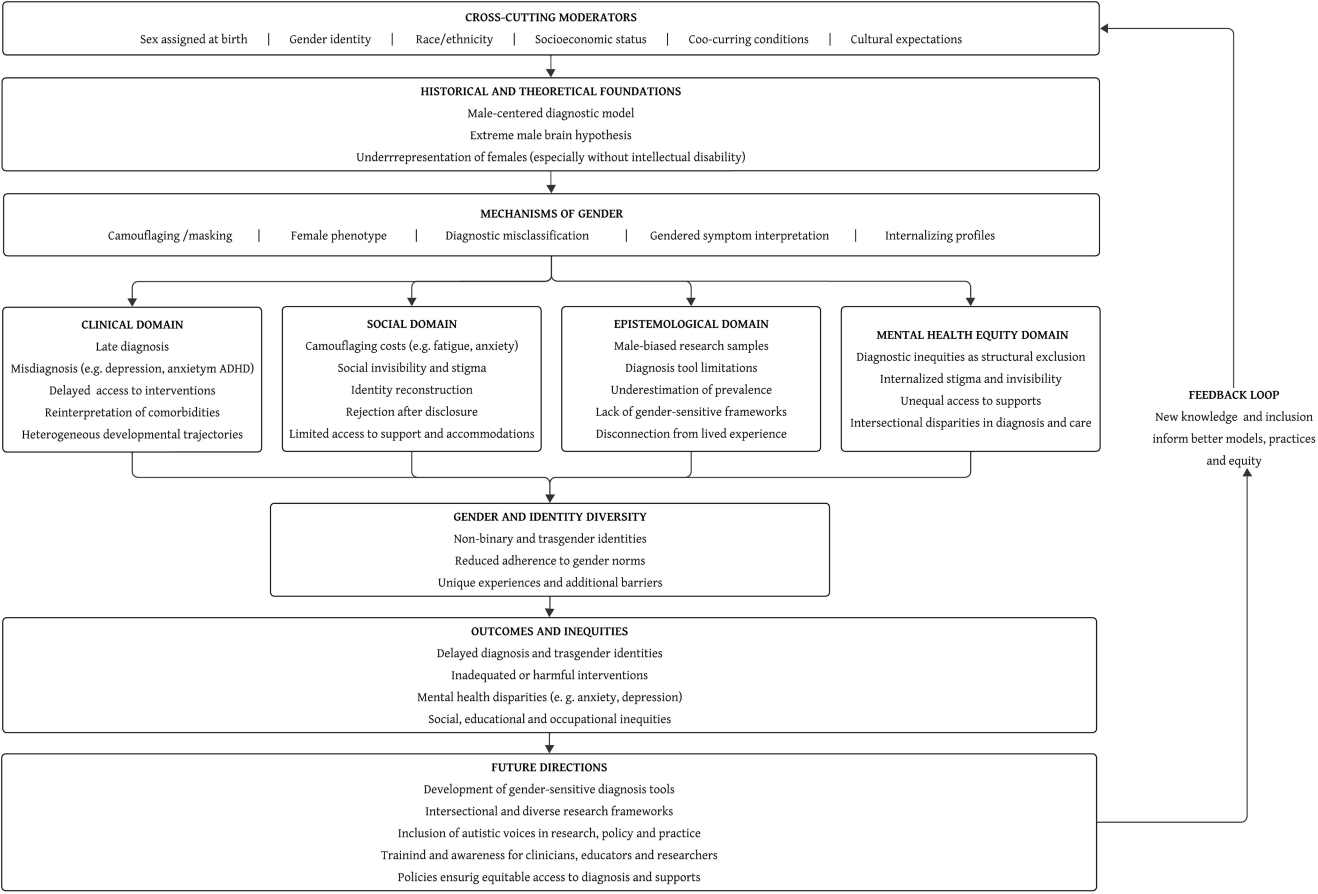
Conceptual framework of gender bias in autism research and diagnosis.

The literature reveals a set of intertwined clinical, social, epistemological, and equity-related challenges that shape current understandings of autism, as summarized in **Figure 1**, which offers a visual map of the overall flow of the argument.

### Clinical domain

The study of gender differences in the manifestation and diagnosis of autism goes beyond mere academic interest, highlighting a persistent gap in mental health.

Building on these historical foundations, current diagnostic frameworks may still insufficiently capture autistic presentations in women and gender-diverse individuals (7). This has resulted in many individuals being undiagnosed or receiving a late diagnosis, limiting early access to effective interventions (14). Recent evidence supports this concern. Aizenberg et al. (45) identified two profiles among those receiving a late diagnosis: one with less intensive support needs, and another characterized by multiple prior psychiatric misdiagnoses before reaching an autism diagnosis. The latter group, composed primarily of women, highlights how autistic symptoms can be mistaken for depression (46), anxiety (47) or Attention Deficit and Hyperactivity Disorder (48). Indeed, several studies confirm this trend. Kentrou et al. (49) showed that autistic adults often report previous misdiagnoses of mood disorders, anxiety, or personality disorders. These diagnostic trajectories appear to be particularly common among women, whose autistic traits may be interpreted as symptoms of other psychiatric conditions before an autism diagnosis is considered. Similarly, McQuaid et al. (50) demonstrate the frequent confusion between autistic traits in women and borderline personality disorder, which significantly delays adequate intervention. Downs and Adams (51) underscore how late diagnosis can substantially reshape the understanding and treatment of other conditions, such as eating disorders, by providing a new comprehension of the underlying source of distress.

This evidence demonstrates that diagnostic confusion not only delays autism detection but can also lead to inadequate or even harmful interventions. This situation reinforces the need to develop gender-sensitive clinical protocols capable of recognizing the diversity of expressions within the autistic spectrum. Overall, this pattern of diagnostic substitution and delayed recognition indicates that current clinical frameworks insufficiently account for gendered expressions of autism, systematically increasing the risk of misdiagnosis and postponing access to appropriate care.

### Social domain

Turning to the social domain, the predominance of a historically male-based framework has contributed to the invisibility of non-male autistic experiences, reinforcing stereotypes and hindering the identification of specific needs. This invisibility heightens the risk of social isolation and restricts autistic women’s access to adequate support across childhood and adulthood, thereby perpetuating social and mental health inequities (52). Numerous studies have further documented the social impact of this invisibility. Bargiela et al. (12) found that many autistic women diagnosed in adulthood develop particularly intense social camouflaging strategies, with consequences such as fatigue, anxiety, and heightened vulnerability to victimization. Similarly, Evans et al. (11) reported that camouflaging is associated with low self-esteem, interpersonal trauma, and difficulties sustaining authentic relationships. Indeed, Garcia-Molina (53) shows that women disclosing their diagnosis in social contexts often face skepticism or rejection from family and friends, which increases feelings of alienation. Bransgrove and Karakas (54) also explored how autistic individuals diagnosed later in life reconstruct their personal identity in environments that frequently fail to validate their condition as a legitimate part of their identity. The lack of external validation and the late recognition of neurodivergence thereby intensify experiences of isolation and self-stigmatization, with long-lasting social and emotional consequences. Collectively, these findings suggest that the social invisibility of autistic women is not merely descriptive but actively produced through camouflaging demands and lack of validation, resulting in cumulative psychosocial disadvantage across the lifespan.

### Epistemological domain

Gender bias in research has distorted epidemiological data, affecting the overall understanding of autism as a human condition (39). This distortion influences the design of diagnostic and intervention models, perpetuating criteria that fail to capture phenotypic diversity. Science thus faces the challenge of redefining diagnostic frameworks to incorporate variations in autistic expression by gender and sociocultural context (55). Along these lines, Ghanouni and Seaker (56) emphasize the disconnection between standardized diagnostic instruments and the narratives of adults diagnosed later in life. The study by Dubreucq et al. (57) reinforces this critique by showing that social camouflaging (particularly among women) predicts a later age of diagnosis, which has direct implications for the design of detection tools. Likewise, Jonkman et al. (58) question the uncritical generalization of interventions derived from Applied Behavior Analysis (ABA), which do not account for autistic expression in adults diagnosed late.

In sum, autism research requires an epistemological transformation capable of incorporating gender, age, life trajectories, and sociocultural contexts, while also systematically distinguishing between sex assigned at birth and gender identity in research design. Taken together, this evidence indicates that current epistemological frameworks not only underrepresent autistic diversity but also actively shape what is clinically recognizable, thereby perpetuating systematic blind spots in diagnosis and intervention research.

### Mental health equity domain

Finally, these findings also have implications for mental health equity. From a human rights perspective, omission or diagnostic disparity constitutes a violation of gender equity in mental health. Diagnostic and therapeutic care must respond to the actual characteristics of the population, rather than stereotypes or biased patterns (59, 60). Addressing this issue is not only a clinical necessity but also an ethical imperative to ensure fair access to care and a reduction in health inequalities.

The work of Huang et al. (61) examines internalized stigma in autistic adults diagnosed later in life, who report having spent years trying to “be normal,” often without understanding their own difficulties. This stigma, often rendered invisible, impacts self-perception and access to adequate support. Frizell (62) suggests that recognizing the diversity of diagnostic trajectories allows autism to be understood not as an isolated pathology but as a complex and multifaceted identity. Additionally, other studies highlight that inequity in access to diagnosis generates not only clinical but also social, educational, and occupational consequences. This inequity prevents many individuals from accessing the supports and accommodations necessary in daily life, education and work (63, 64).

It is noteworthy, however, that despite the fact that gender has been one of the most researched factors related to late diagnosis, suggestions have been made to broaden the focus to other equally relevant elements such as race, socioeconomic status, intelligence, parental mental health, and co-occurring conditions, to name just a few (65). Along these lines, Goldblum et al. (66) showed that the intersection between sex assigned at birth, race, and ethnicity influences the age and quality of diagnosis, widening the equity gap for already vulnerable populations. These intersectional factors not only shape timely access to adequate clinical evaluation but also condition the interpretation of symptoms, often filtered through cultural, racial, and gender stereotypes. As a result, these individuals often face multiple diagnostic barriers, including the underestimation of their difficulties or the misattribution of their behaviors to socio-environmental factors (67). This structural lack of visibility reinforces historical inequalities and underscores the urgent need to rethink diagnostic frameworks from a critical, inclusive, and grounded perspective—one that recognizes the complexity of identities and social contexts (36, 68, 69). In this sense, diagnostic inequities function not only as clinical shortcomings but as structural forms of exclusion that compound across social, educational, and occupational domains, particularly for intersecting marginalized groups.

Across these domains, a consistent pattern emerges: gender and intersectional biases are embedded at multiple levels of knowledge production and clinical practice, jointly contributing to delayed diagnosis, social invisibility, and unequal access to care.

## Current challenges and future directions

The transformation required is both structural and epistemological. One of the most urgent issues is the reformulation of diagnostic tools, which remain rooted in a male-centered model of autism. This reformulation may involve revising the normative samples used to standardize diagnostic instruments, reconsidering behavioral and cognitive thresholds, incorporating indicators of social camouflaging, and adapting clinical interpretation guidelines and cut-off scores to better capture gender-related differences in autistic presentations.

As recent studies show (41), conventional assessments fail to capture the female profile and the camouflaging strategies typical of women and gender-diverse individuals, delaying diagnosis and limiting access to appropriate support.

Among the most pressing challenges is the need to promote intersectional research that incorporates variables such as gender, age, ethnicity, social class, and neurodiversity. The systematic review by Russell et al. (15) and the findings of Goldblum et al. (66) demonstrate that current frameworks systematically underestimate the true prevalence of autism in women, individuals of different races, and historically marginalized groups. This disparity is amplified when diagnostic tools are applied uncritically across culturally diverse contexts. For instance, behaviors interpreted as atypical social communication within some diagnostic frameworks—such as reduced eye contact or reserved social interaction—may be considered culturally normative in other societies. Such differences can influence both the recognition of autistic traits and the likelihood of referral for diagnostic assessment.

It is also essential to actively include autistic people in the design, implementation, and validation of scientific studies. Ghanouni and Seaker (56) and Frizell (62) emphasize that lived narratives and experiential knowledge are fundamental for generating diagnostic and therapeutic models that are more representative and sensitive. To reach true co-design, such participation must go beyond a merely consultative role, extending to decision-making positions in methodological and ethical aspects of knowledge production.

Finally, the field must move toward a more inclusive and representative spectrum model, one that transcends dichotomous or linear approaches. This requires rethinking diagnostic categories and recognizing the diversity of trajectories, manifestations, and needs within the spectrum.

## Conclusions

Gender bias in autism diagnosis has historically been normalized and has only recently, within the last two decades, begun to be recognized as problematic. From the earliest androcentric diagnostic model to diagnostic systems built on non-representative samples, a partial and exclusionary view has been consolidated—one that has marginalized women, non-binary individuals, and intersectionally vulnerable groups (70).

Recognizing and examining these differences is essential to advancing diagnostic justice and mental health equity. For example, autistic women may present subtler social communication differences, restricted interests that appear socially typical, greater reliance on compensatory social strategies, and higher levels of internalizing symptoms such as anxiety or depression. Non-binary or gender-diverse autistic individuals may also experience unique challenges related to social expectations and identity development, which can influence both symptom expression and pathways to diagnosis.

The reviewed evidence shows that underdiagnosis not only restricts access to support, but also leads to consequences such as stigmatization, identity confusion, inadequate treatments and the loss of educational, occupational and relational opportunities (12, 53).

Addressing gender bias in autism therefore requires more than refining diagnostic tools. It calls for a broader paradigm shift in how autism is conceptualized, researched, and assessed. Future clinical and scientific frameworks should systematically consider sex assigned at birth, gender identity, camouflaging, developmental trajectories, and sociocultural context, while also incorporating the lived experience of autistic people in the production of knowledge. Moving toward a more inclusive understanding of autism is not only scientifically necessary; it is an ethical imperative for achieving genuine diagnostic justice and mental health equity.

## Data Availability

The original contributions presented in the study are included in the article/supplementary material. Further inquiries can be directed to the corresponding author.

## Footnotes

1 This work employs both person-first language (e.g., “person with autism”) and identity-first language (e.g., “autistic person”) in order to respect the diverse preferences within the autistic community. Medical terminology (ASD) is used solely when referring to diagnostic criteria or clinical contexts.
